# Functional Abilities as a Predictor of Specific Motor Skills of Young Water Polo Players

**DOI:** 10.2478/v10078-011-0046-5

**Published:** 2011-10-04

**Authors:** Marko Aleksandrović, Dragan Radovanović, Tomislav Okičić, Dejan Madić, Georgi Georgiev

**Affiliations:** 1 Faculty of Sport and Physical Education, University of Niš, Niš, Serbia; 2 Faculty of Physical Education, University of Skopje, Skopje, FYR Macedonia

**Keywords:** water polo, youth athletes, functional abilities, swim test

## Abstract

The purpose of this study was to assess the influence of functional abilities on specificmotor skills. A total number of 92 male water polo players (age 12±0.5 years, body height 156.96±22.3 cm, body weight 51.02±33.18 kg) with at least two years’ experience, were enrolled in the study. The investigation protocol consisted of standardized anthropometric measurements, estimation of maximum oxygen uptake, determination of the lung function values, specific swim tests and swim tests with a ball. The factor analysis was used for the estimation of the structure of specific motor skills. The influence of functional abilities on specific motor skills was estimated by regression analysis. Out of 15 correlations in total between the variables of space of functional abilities of water polo players, 6 were significant at the level of 95% (between the variables of aerobic power and lung function) and all of the correlations (15) between the variables of specific motor skills in water polo players were significant at the 99% level. Only one principal component, the General factor of specific motor skills in water polo (GFSWP) was obtained by way of factorization of the tests of specific motor skills, so the GFSWP represents the latent space of specific motor skills as a criterion. The regression analysis showed that functional abilities (as group predictors) (p= 0.00) and forced expiratory volume in 1 second (as a separate variable) have a significant influence on GFSWP (the criterion). The results of the study pointed out the impact of functional abilities on specific motor skills of selected young water polo players. This may be important for the selection and effective coaching in the early period of training and can affect the development of more appropriate and specific training programmes for optimal physical fitness preparation in young water polo players.

## Introduction

Water polo is a complex and physically demanding sport, composed of high intensity bursts of sprinting, interspersed with short periods of low to moderate intensity swimming. During the game, water polo players take one of two basic swimming positions – the horizontal or the vertical one – from which they realize various technical and tactical (TE-TA) activities. A water polo player spends from 33.1% to around 45% of time in various variants of horizontal position, and from 55% to 66.9% in various options of the vertical position from which he realizes his TE-TA tasks (Dopsaj and Thanopoulos, 2006; Dopsaj and Aleksandrović, 2009; Pinnington et al., 1988; Smith, 1998). These characteristics of water polo require adequate conditioning, involving both specific motor skills and functional abilities (Pavlik et al., 2001a; Pavlik et al., 2001b; Platanou, 2009; Smith, 1998). It has been known that high performance in various sports is generally the result of long-term training build-up. A build-up period is divided into different smaller units, each with different goals. Water polo players begin their regular physical training in early childhood (Horvath et al., 2009; Varamenti and Platanou, 2008). There are periods when the development of some abilities is accelerated, followed by periods of slower development. Reaction speed and frequency of movement are the abilities with a dynamic development in the period from 7 to 11 or 12 years of age (Armstrong et al., 2001). The development of endurance changes unevenly, yet with an increasing tendency. The period of puberty is thought to be very sensitive for the development of aerobic and anaerobic endurance (Armstrong and Welsman, 2000). Pulmonary function develops and increases with age. Pulmonary variables increase until physical maturation is reached. These increases are directly related to body growth (Bailey et al., 1995). The functional indicators of the cardiovascular system are significantly dependent on body mass. Children have lower stroke volume and blood pressure, but a higher heart rate compared to adults. Younger children are more prone to tachycardia and tachypnea during physical exercise, since they adapt their small cardiorespiratory potential to the given level of stress (Rowland et al., 1997). At submaximal levels of exercise, arteriovenous difference in oxygen in children is higher than in adults, compensating for lower stroke volume of the heart (Baxter-Jones et al., 1993). Improvement of pulmonary and cardiovascular function during growth produces an increase of aerobic capacity (Armstrong and Welsman, 1994).

Serbia and former Yugoslavia have a great water polo tradition (3 times Olympic gold medal winners, 4 times World champions, 4 times European champions, 4 FINA Cup gold medal winners, 4 times FINA World League champions, etc.) in men’s water polo. This very successful and demanding water polo school requires an early start in order to be able to produce top results in the future. It is necessary to start training in the tenth year of life, so that young 12 year old players already have competition experience at the national level, knowledge of TE-TA, and a high level of swimming abilities (Aleksandrović et al., 2007).

Water polo has not been studied enough, probably because of the limited competition and the difficulties related to data collection under water (Platanou, 2009), despite its rich history and rapid evolution (Donev and Aleksandrović, 2008). Information related to the physiologic abilities of water polo players is scarce, as well as the information about their impact on specific-motor skills (Aleksandrović et al., 2004; Platanou, 2009). Also, there are less data about such problems at the first level of water polo selection. The aim of our study was to assess the impact of functional abilities on specific motor skills of young water polo players.

## Material and methods

The studied sample was composed of 92 young water polo players from four clubs, aged 12 ± 6 months, body height 156.96 ± 22.32 cm, body mass 51.02 ± 33.18 kg, with at least two years of training and competition experience. All of the subjects were involved in a water polo training program (15 minutes of stretching, prior to 60–75 minutes of specific training, 3–4 times a week) designed to improve swimming technique and to master technical elements of water polo. Moreover, the athletes were included in a competition system with more than 15 matches per season.

All the athletes, their parents and coaches were given the necessary information about the objectives, course, participation and possible side-effects of the study. All examined subjects underwent general physical evaluations before the enrollment. The whole study protocol was executed in the presence of parents and/or coaches. None of the study participants had any anamnestic information or clinical finding of physical exercise-induced bronchoconstriction.

Anthropometric measurements were conducted according to the conventional criteria, ethical restrictions and procedures using an anthropometer (GMP, Switzerland). Functional investigations started with lung function (forced vital capacity – FVC and forced expiratory volume in 1 second – FEV_1.0_) measurements. All subjects reported a negative history of clinically diagnosed exercise-induced bronchoconstriction. Subjects were required to perform FVC maneuvers on a computerized spirometer according to the American Thoracic Society standardization of spirometry. Maximum oxygen uptake (VO_2peak_) was registered utilizing the extrapolation method (American College of Sports Medicine, 2006) after a standardized submaximal test on a cycle ergometer (Kettler, Germany), along with telemetric monitoring of heart function (Polar, Finland). The testing was carried out at least 24 hours prior to the swimming tests. All laboratory testing was carried out in the morning hours, in a room with a temperature of 21–23°C and humidity 55–60%, to fulfill the standards for functional laboratory testing (Winter et al., 2006).

### Swimming tests

On the day of testing, after 10 minutes of standardized swimming warm-up, the subjects performed swimming tests (long course swimming pool, depth 2.1 m and the water temperature 27.5°C): 25 m water polo crawl (SW25), 50 m crawl (SW50) and 100 m crawl (SW100) (Volčanšek & Grčić-Zubčević, 1984). Each of the subjects had two attempts and the better one was taken into consideration. For motivation purposes, the subjects were immediately informed about the results (Dopsaj et al., 2007). The day after these evaluations, all subjects performed the following specific tests: Swimming 4 × 5 m front and back crawl (SW4x5), Leading the ball in water 3 × 5 m (SW3x5) and Throwing the ball from the water (TBall). Both tests with a ball were performed with the official ball for the age category of our study participants (Tigar Mini, Serbia).

The 25 m water polo crawl (SW25) with the head above water, with athletes starting in the water with the feet on the wall. The 50 m crawl (SW50) and 100 m crawl (SW100) were performed according to FINA rules. All three swimming tests were measured by an electronic system (Alge Swim 2000, Austria). The day after these evaluations, the subjects performed the following tests: Swimming 4 x 5 m front and back crawl (SW4x5), Leading the ball in the water 3 x 5 m (SW3x5) and Throwing the ball from the water (TBall) (Bratuša, 2000).

Swimming 4 × 5 m front and back crawl (SW4x5). Two floating lanes were placed in the water opposite to one another at 5 m distance. The athletes were in the water with the head in front of the first floating lane. After a signal of the coach, the athlete started to swim water polo crawl to the opposite floating lane, touching it and turning back with a backstroke. After the second turn, the examinee changes his technique into water polo crawl, as well as after the third turn, once again with a backstroke. The test ended when the subject for the second time finished the backstroke distance.

Leading the ball in the water 3 × 5 m (SW3x5). Two floating lanes were placed in the water opposite to one another at 5 m distance. The examinee was in the water with the head in front of the first floating lane, and the ball was in front of the head. After a signal of the coach, the athlete started to swim with the ball in front of the head to the opposite floating lane, touching it, and swimming back with the ball. The test ended when the subject for the third time finished the 5 m distance between the floating lines. For these two mentioned water polo tests, the times were recorded manually. Three referees, experienced national water polo coaches, used a stopwatch. The mean time was used for evaluation purposes.

Throwing the ball from the water (TBall). The subject was in the water with the ball in his hand. The floating line was in front of his head, representing the starting point for throwing distance calculation. Along the pool, there were marked points at every meter. The athlete was asked to throw the ball as far as he could. The results were calculated with 0.5 m accuracy by experienced national water polo referees. The better of the two results was used for calculations.

### Statistical procedures

In order to establish the level of examined functional abilities and specific motor skills of water polo players, some basic descriptive statistic parameters were established (Mean – arithmetic mean, SD – standard deviation, CV% – coefficient of variability, Min – minimum value, Max – maximum value, KS – Kolmogorov-Smirnov test, Scew – skewness, Kurt – kurtosis).

The correlations (r) between the variables of space of functional abilities, as well as of specific motor space of water polo players, were calculated (significant correlation coefficients were at the 95% level as well as at the 99% level, high correlations existed if r≥ 0.70).

The structure of specific motor skills was established utilizing the component factor analysis – the Hotelling’s method of principal components with the GK criterion (the characteristic root Lambda of ≥1.00 was considered) with six manifested variables. Total variability in the system of utilized variables (Percent) was calculated, as well as the communalities of the variables of specific motor skills (h^2^). The number of obtained principal components when Lambda was ≥1.00 would be considered as the representative criterion of latent space of specific motor skills for linear regression analysis.

The impact of functional abilities on motor skills was assessed utilizing linear regression analysis (R – Pearson’s coefficient of correlation Part-R – Partial correlations, BETA – standardized beta coefficient, t(86) – degrees of freedom, P – level of significance, significant if the value was ≤.05, Rmc – multiple correlation coefficient, R^2^ – coefficient of determination, p – level of significance of the predictor system on the criterion).

## Results

The mean values indicated good discrimination of the measurements. Regarding variability, SD in all the observed variables was contained at least thrice in the mean of the results (Mean). SDs of these tests were adequate, demonstrating sufficient sensitivity. The same can be said for CV%, the results of which are within the accepted limits (below 25), indicating an outstanding homogeneity of the studied sample of players. The KS indicated normal distribution of the results (below 1.00). The skewness was adequate for the population of selected water polo players. The obtained results were widely spread, indicating platycurtic distribution. The studied sample of water polo players was homogenous regarding their functional abilities and specific motor skills (Table 1).

The correlations between the variables of space of functional abilities of water polo players (Table 2) were significant if the correlation coefficients were relatively high: from r= 0.21 at the 95% level and r=0.27 at the 99% level. Out of 15 correlations in total, 6 were significant at the level of 95%. One very high correlation was located (r≤ 0.70) among the lung function variables (r(FVC/FEV)= .73). Intercorrelations were present between the variables of aerobic power and lung function (AVO_2peak_, RVO_2peak_, FVC, FEV_1.0_). HRrest did not have any significant correlations with other considered variables.

Correlations between the variables of specific motor skills space in water polo players (Table 3) were significant in cases with higher correlation coefficients r= 0.27 at the level of 99%. Out of 15 correlations in total, all were significant at the level of 99%. Individually, high intercorrelations in the situational-motor skills in water polo players (r≤ 0.70) were found only for the SW50 variable ((r(SW50/SW25)) = .72, r((SW50/SW100)) = .84).

Only one principal component, the General factor of specific motor skills in water polo (GFSWP) was obtained by means of factorization of specific motor skills tests of water polo players utilizing the Hotelling’s method of principal components with the GK criterion (characteristic root of ≥1.00 was observed) with six manifested variables. Only one isolated statistically significant root (Lambda) was of the value 3.83, explaining the total variability in the system of utilized variables (Percent) with 63.85%. Variable projections to the principal component (GFSWP) were bipolar, with absolute values ranging from .65 (SW4x5) to .91 (SW50). The communalities of the variables of specific motor skills (h^2^) were moderately high (.52 do .83), except for the SW4x5 variable, with a lower communality value (.42), probably because of more pronounced measurement errors.

The only one isolated factor of significance, GFSWP, will be used in the regression analysis as the criterion to establish associations between the tudied functional abilities (a predictor system).

The association of the whole predictor system of variables of functional abilities and GFSWP was relatively high (R= .46). Common variability between the predictor system and criterion variable was around 21% (R^2^= .21), of significance at the level of 99% (p= .00). The remaining 79% in the explanation of total GFSWP variability can be attributed to other abilities and characteristics of the athletes, left out in this regression analysis. Individually, the FEV variable had a significant impact on GFSWP (BETA= − .29), which was significant at the level of 95%.

## Discussion

The present study provided the information on functional abilities of young water polo players and their impact on specific motor skills. The results presented in Table 1 show higher values of body height and body mass, functional and specific motor abilities of water polo players, compared to their peers not practicing any sport, which was also recorded in some past studies with the sample of players of identical age (Lupo et al., 2009; Radovanović et al., 2004). The differences in body height can be explained by a direct selection for water polo. Body mass differences can be considered as consequence of training, direct selection, nutrition and longer periods of time spent in the water. Since the athletes were 11–12 years old, these variables and differences between them were going to change significantly during puberty. The obtained values of maximal oxygen uptake in our study (48.09 ± 9.85 mL × kg^−1^ × min^−1^) were approximately the same as the values of young water polo players of the same age (47.9 ± 2.9 mL × kg^−1^ × min^−1^) estimated by a one-mile walk test (Lupo et al., 2009). Smith (1998) has claimed that aerobic capability is essential in the improvement of players’ ability to recover from high-intensity anaerobic efforts during matches. The presented VO_2peak_ values indicated that water polo training had a beneficial effect on aerobic capacity in children.

Table 2 presents the data of the correlation matrix of variables of functional abilities of water polo players, illustrating the correlation between maximum oxygen consumption and lung function parameters (AVO_2peak_, RVO_2peak_, FVC, FEV_1.0_), being the consequence of long-term alternations of long intervals of physiologic dormancy with game intervals during water polo training (Marrin and Bampouras, 2008; Platanou, 2009; Smith, 1998). These findings supported our assumption that intense training in a sports discipline with both aerobic and anarobic demands, induces increased cardiorespiratory endurance very early, even in puberty. We should perhaps add that there is no consensus regarding the optimum intensity, frequency, or duration of physical activity leading to increased cardiorespiratory endurance in prepubertal children (Nevill et al., 1998). Earlier studies had even questioned the possibility of increasing cardiorespiratory endurance in children in view of the difficulties associated with achieving and maintaining the intensity and duration of planned training and the usual physical activity in that period (Baxter-Jones and Maffulli, 2003). Physiologic adaptation to training, expressed by way of significant increase of absolute values of VO_2peak_, successfully compensates for the increased body mass of the examinees. In other words, the children participating in water polo training have an increased ability to sustain longer physical acitivity, involving relatively large muscle groups, regardless of an increased body mass. The results of similar studies have demonstrated that longitudinal skeletal dimension is proportional to the swimming velocity and to success in water polo later on.

The results presented in Table 3 show the data of the correlation matrix of variables of specific motor abilities of water polo players, demonstrating a correlation of utilized tests, though the battery of tests was composed of both polycyclic anaerobic-lactic (SW25, SW50, SW100, SW4x5, SW3x5) and acyclic monostructural anaerobic-alactate tests (TBall). The explanation of these facts could be sought in the fact that the subjects in this study were engaged in a process of specific training for two years, with combined aerobic and anaerobic training loads. In similar studies, Rechichi et al. (2000) concluded that the 10 m multistage shuttle swim test could be a reliable and valid field test of aerobic fitness for use with trained water polo players, while Garrido et al. (2010) suggested that simple dry land strength and power tests, although moderate, were significantly associated with sprint swimming performance in young competitive swimmers. Earlier, Swaine and Zanker (1996) established the reproducible assessments of cardiopulmonary responses to exercise using an isokinetic swim bench for highly trained swimmers.

Table 4 shows the factorization results of the specific motor abilities tests of water polo players using the Hotelling’s principal component method. Only one isolated principal component, i.e. the GFSWP factor, is the consequence of a selected homogenous sample and appropriate selection of tests in a battery. We believe that the applied battery of tests can be utilized to diagnose specific motor abilities in water polo players of that age, and that the results can serve as a determinant in training planning. At the same time, sample homogeneity regarding the specific motor abilities is the evidence of adequate training, since in most of the athletes good command of water polo specific skills was observed.

Regression analysis results in Table 5 demonstrated a relatively high association of all the variables of functional abilities with GFSWP, indicating that the applied tests of functional abilities allowed for the assessment of success in the battery of specific tests of the motor abilities, which had been confirmed in earlier studies of the sample of 14–15 year old water polo players (Aleksandrović et al., 2004). A summary impact of the parameters of pulmonary function, maximum oxygen uptake and heart rate at rest on the exercise of specific motor skills was the consequence of a long-term training process. In the water polo players with years-long sports experience, there is the potential of their heart function to adapt more rapidly to challenging events and they also have a lower heart rate at rest (Okičić, 1999; Pavlik et al., 2001a; Pavlik et al., 2005), leading even to athletic bradycardia in top water polo players (Petridis et al., 2003; Zakynthinos et al., 2001) and high values of VO_2peak_ and pulmonary function parameters in their senior sports age (Colantonio et al., 2003; Frenkl et al., 2001; Geladas and Platanou, 2000; Horváth et al., 2009; Marrin and Bampouras, 2008; Pavlik et al., 2001b; Pavlik et al., 2005; Platanou et al., 2009; Smith, 1998; Tan et al., 2009; Tsekouras et al., 2005).

Knowledge of the impact of functional abilities on specific motor skills in young water polo players is important for selection and effective coaching in early period of training and will facilitate the development of more appropriate and specific training programmes for optimal physical preparation. Although studies are not always in agreement as to the importance of each of the aspects, it can be argued that they, if measured appropriately, may provide coaches with relevant information on the young prospect’s natural ability (Falk et al., 2004). The applied battery of tests can be considered appropriate to assess the specific motor abilities of water polo players. Further studies of youth water polo training adaptations could provide coaches with more thorough understanding of the development of specific motor skills and possibilities of their improvement.

Although a relatively large sample of selected young players and the selection of the battery of tests (testing a large number of functional and motor abilities) are an advantage of the conducted study, there are yet certain limitations. The main limitation of our study was that the differences in tests, protocols and equipment, allowed only for limited comparisons of the obtained data with other water polo studies reported in the literature.

## Figures and Tables

**Table 1:** Basic statistic parameters of body height and body mass, functional abilities, and specific motor skills of water polo players

Variable (unit)	Mean	SD	CV%	Min	Max	KS	Skew	Kurt
Body height (cm)	156.98	8.36	5.33	136.00	179.30	.99	.23	.19
Body mass (kg)	51.02	11.19	21.93	31.20	85.20	.30	.64	.47
AVO_2peak_ (L x min^−1^)	2.41	.42	17.43	1.60	4.00	.03	1.01	2.14
RVO_2peak_ (mL × kg^−1^ × min^−1^)	48.09	9.85	20.48	25.04	81.63	.98	.38	.51
FVC (L)	3.73	.68	18.23	2.23	5.80	.33	.42	.24
FEV_1.0_ (L)	3.15	.44	13.97	2.22	4.56	.82	.23	.35
HRrest (beats × min^−1^)	78.07	10.54	13.50	60.00	100.00	.07	.25	−.98
SW25 (s)	18.05	1.80	9.97	14.60	22.80	.14	.63	− .16
SW50 (s)	38.81	4.46	11.49	30.95	51.45	.18	.77	.38
SW100 (s)	85.90	9.73	11.33	63.40	113.00	.10	.66	.18
SW4x5 (s)	22.15	2.04	9.21	16.88	27.33	.11	.30	.40
SW3x5 (s)	16.31	1.64	10.06	12.64	21.07	.49	.81	.89
TBall (m)	14.89	2.82	18.94	7.00	24.50	.04	.37	1.28

**Mean** – arithmetic mean, **SD** – standard deviation, **CV%** – coefficient of variability, **Min** – minimum value, **Max –** maximum value, **KS** – Kolmogorov-Smirnov test, **Scew** – skewness, **Kurt** – curtosis, **AVO**_2peak_ – Absolute values of maximum oxygen uptake, **RVO**_2peak_ – Relative values of maximum oxygen uptake, **FVC** – Forced vital capacity, **FEV_1.0_** – Forced expiratory volume in 1 second, **HRrest** – Heart rate at rest, **SW25 –** 25m water polo crawl, **SW50** – 50m crawl, **SW100** – 100m crawl, **SW4x5** – Swimming 4x5m front/back crawl, **SW3x5** – Leading the ball in water for 3x5m, **TBall** – Throwing the ball from the water.

**Table 2:** Correlation matrix of the variables of functional abilities of water polo players

				
Variables	AVO_2max_				
			
AVO_2max_	1.00	RVO_2max_			
				
RVO_2max_	r= 0.43^**^	1.00	FVC		
				
FVC	r= 0.35^**^	r= − 0.27^**^	1.00	FEV_1.0_	
				
FEV_1.0_	r= 0.25^*^	r= −0.21^*^	r= 0.73^**^	1.00	HRrest
					
HRrest	ns	ns	ns	ns	1.00

**AVO**_2peak_ – Absolute maximum oxygen uptake, **RVO**_2max_ – Relative maximum oxygen uptake, **FVC** – Forced vital capacity, **FEV**_1.0_ – Forced expiratory volume in 1 second, **HRrest** – Heart rate at rest.

* or ** Represents significance for p≤0.05 and p≤0.001, respectively; ns, represents the non-existence of significance.

**Table 3:** Correlation matrix of the variables of specific motor skills of water polo players

					
Variables	SW25					
				
SW25	1.00	SW50				
					
SW50	r= 0.72^**^	1.00	SW100			
					
SW100	r= 0.66^**^	r= 0.84^**^	1.00	SW4x5		
					
SW4x5	r= 0.33^**^	r= 0.48^**^	r= 0.39^**^	1.00	SW3x5	
					
SW3x5	r= 0.67^**^	r= 0.68^**^	r= 0.60^**^	r= 0.50^**^	1.00	TBall
						
TBall	r= −0.44^**^	r= −0.56^**^	r= −0.49^**^	r= −0.52^**^	r= −0.50^**^	1.00

**SW25** – 25m water polo crawl, **SW50** – 50m crawl, **SW100** – 100m crawl, **SW4x5** – Swimming 4x5m front/back crawl, **SW3x5** – Leading the ball in the water 3x5m, **TBall** – Throwing the ball from the water.

** Represents significance for p≤0.001.

**Table 4:** Factorization of tests of specific motor skills of water polo players utilizing the Hotteling’s method of principal components and their communalities

Variables	Projections	h^2^
SW25	0.81	0.66
SW50	0.91	0.83
SW100	0.85	0.72
SW4x5	0.65	0.42
SW3x5	0.83	0.69
TBall	− 0.72	0.52

Lambda	3.83	

Percent	63.85	

**SW25 –** 25m water polo crawl, **SW50** – 50m crawl, **SW100** – 100m crawl, **SW4x5** – Swimming 4x5m front/back crawl, **SW3x5** – Leading the ball 3x5m, **TBall** – Throwing the ball from the water. **h**^2^**–** communalities, **Lambda –** characteristic root, **Percent** – % of explained variance.

**Table 5:** Results of the regression analysis of functional abilities and GFSWP

Variables	R	Part R	BETA	t(86)
AVO_2peak_	−0.35	−0.17	−0.20	−1.59
RVO_2peak_	−0.10	−0.07	−0.08	−0.67
FVC	−0.33	−0.04	−0.05	−0.38
FEV_1.0_	−0.36^*^	−0.22	−0.29	−2.08
HRrest	0.13	0.09	0.08	0.81

	Rmc= 0.46^**^	R^2^= 0.21		

**AVO**_2peak_ – Absolute maximum oxygen uptake, **RVO**_2peak_ – Relative maximum oxygen uptake, **FVC** – Forced vital capacity, **FEV**_1.0_ – Forced expiratory volume in 1 second, **HRrest** – Heart rate at rest. **R** – Pearson’s coefficient of correlation between predictor variables and GFSWP, **Part-R** – Partial correlations between predictor variables and GFSWP, **BETA** – standardized beta coefficient, **T (86)** – degrees of freedom, **Rmc** – multiple correlation coefficient, **R²** – coefficient of determination.

* or ** Represents significance for p≤0.05 and p≤0.001, respectively; ns, represents the non-existence of significance.
